# One Puncture, Two Solutions: Simultaneous Carotid and Iliac Artery Stenting

**DOI:** 10.7759/cureus.66883

**Published:** 2024-08-14

**Authors:** Yukinori Takase, Tatsuya Tanaka, Hirofumi Goto, Nobuaki Momozaki, Akira Matsuno

**Affiliations:** 1 Department of Neurosurgery, Kouhoukai Takagi Hospital, Okawa, JPN; 2 Department of Neurosurgery, International University of Health and Welfare, Narita Hospital, Narita, JPN; 3 Department of Neurology, Imari Arita Kyoritsu Hospital, Arita, JPN; 4 Department of Neurosurgery, Imari Arita Kyoritsu Hospital, Arita, JPN

**Keywords:** endovascular treatment, femoral artery puncture, percutaneous transluminal angioplasty and stenting, carotid artery stenting, iliac artery stenosis, lower extremity artery disease, carotid artery stenosis

## Abstract

An 81-year-old man with asymptomatic severe carotid artery stenosis and symptomatic iliac artery stenosis underwent simultaneous carotid artery stenting (CAS) and iliac artery percutaneous transluminal angioplasty and stenting. The procedure involved transfemoral access, balloon angioplasty, and stenting of the right iliac artery, followed by CAS of the right carotid artery. Similar procedures were performed later on the left iliac and carotid arteries. The patient was discharged with no neurological deficits and remained asymptomatic at a six-month follow-up. Simultaneous CAS and iliac artery stenting were feasible and effective in patients with concurrent severe carotid and iliac artery stenosis, providing a comprehensive revascularization strategy for patients with complex atherosclerotic disease.

## Introduction

Carotid artery stenosis frequently coexists with lower extremity artery disease (LEAD). Studies have shown that 14%-19% of patients with LEAD exhibit significant carotid artery stenosis [1,2]. Carotid artery stenting (CAS) is commonly utilized as an effective intervention for severe carotid artery stenosis in individuals with high cardiovascular risk [3,4]. Although transfemoral access is typically preferred, anatomical challenges, such as aortoiliac occlusive disease or vascular tortuosity, may necessitate alternative access routes, including transcarotid, transradial, or transbrachial approaches [5-9]. This case report shows the successful simultaneous performance of CAS and iliac artery percutaneous transluminal angioplasty and stenting (PTAS), highlighting the comprehensive revascularization strategy used for patients with complex atherosclerotic disease.

## Case presentation

An 81-year-old male presented with progressive carotid artery stenosis and intermittent claudication and was referred to our hospital. His medical history was hypertension, dyslipidemia, angina pectoris, atrial fibrillation, asymptomatic carotid artery stenosis, LEAD, and cellulitis of the lower limb. He had a smoking history, consuming 60 cigarettes per day from the age of 20-45. At the time of presentation, he was on clopidogrel 75 mg/day and apixaban 5 mg/day.

**Figure 1:**
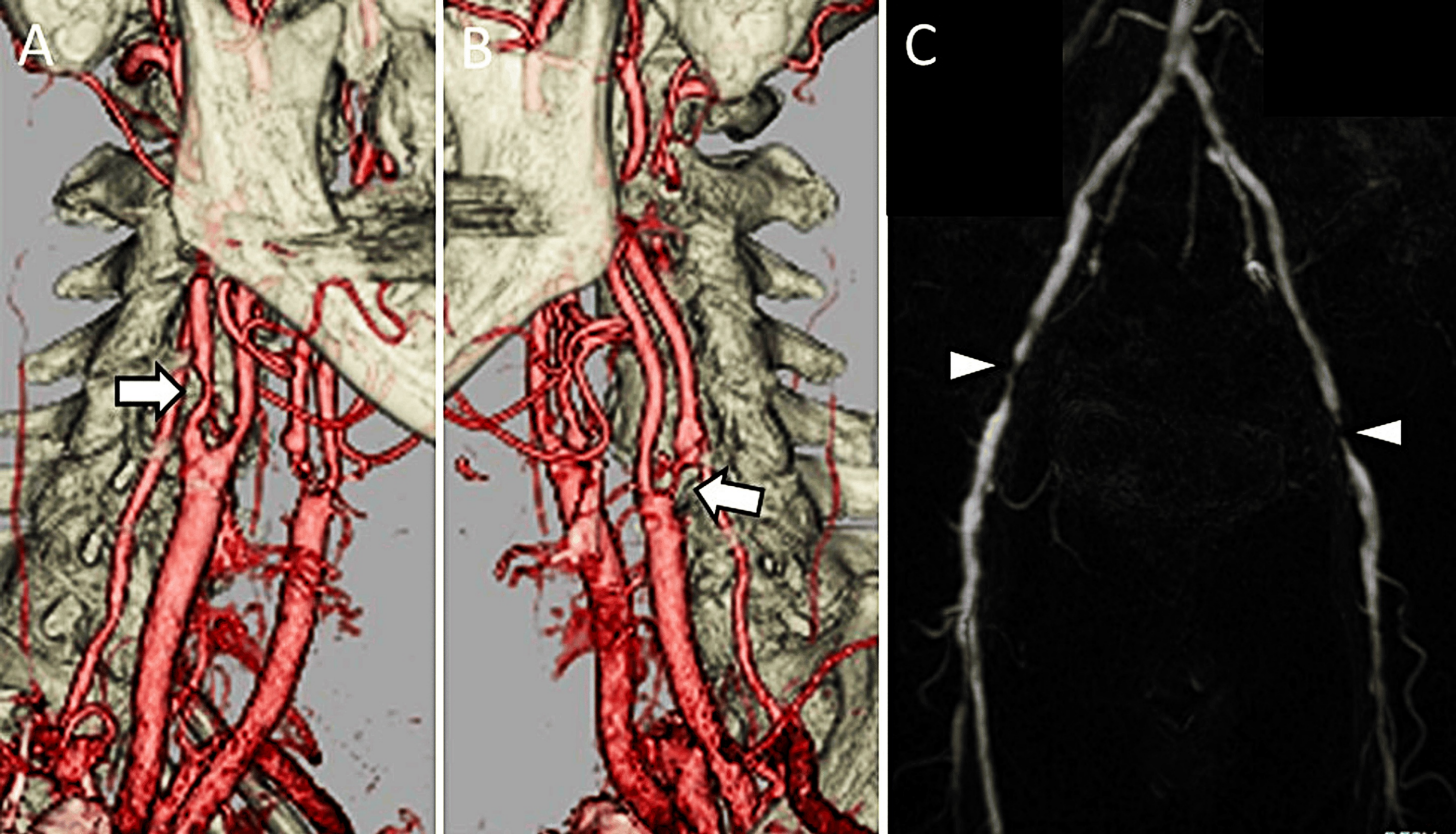
Preoperative images. (A,B) Cervical three-dimensional computed tomography angiography shows bilateral internal carotid artery stenosis (arrows). (C) Lower extremity magnetic resonance angiography demonstrates bilateral external iliac artery stenosis (arrowheads)

On admission, his vital signs were as follows: pulse rate 83 beats/minute, blood pressure 146/68 mmHg, SpO_2_ 98% on room air, and body temperature 36.1°C. The patient was alert with a Glasgow Coma Scale score of 15, exhibited no neurological deficits, and had diminished bilateral dorsalis pedis pulses. Carotid artery ultrasound and cervical three-dimensional computed tomography angiography revealed 88% stenosis of the right internal carotid artery and 95% stenosis of the left internal carotid artery based on the North American Symptomatic Carotid Endarterectomy Trial criteria (Figures 1A, 1B) [10].

The ankle-brachial pressure index (ABPI) was 0.50 and 0.41 on the right and left, respectively. Lower extremity magnetic resonance angiography identified stenoses in both external iliac arteries (Figure 1C). The patient was diagnosed with asymptomatic severe carotid artery and bilateral external iliac artery stenoses.

Due to the patient’s history of heart disease and the risk of thrombosis from discontinuing anticoagulant drugs, CAS was offered along with concurrent endovascular treatment (EVT) of the lower extremity. The decision was made to perform PTAS of the right iliac artery followed by CAS of the right carotid artery and later PTAS of the left iliac artery followed by CAS of the left carotid artery.

**Figure 2:**
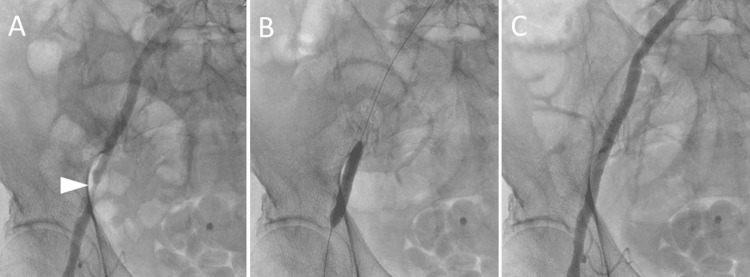
Right iliac artery percutaneous transluminal angioplasty with stenting. (A) Angiography shows stenosis in the right external iliac artery (arrowhead). (B) Stent placement and postdilatation with a balloon. (C) The stenosis of the right external iliac artery is shown to be improved

Digital subtraction angiography (DSA) via transfemoral access showed 75% stenosis of the right external iliac artery. A 6.5-Fr short sheath was introduced into the right femoral artery, followed by passage of a 150-cm guidewire M (Radifocus; Terumo Medical Corporation, Tokyo, Japan) through the stenosis. Intravenous heparin was administered to achieve an activated coagulation time of approximately 300 seconds. Angioplasty of the lesion was performed using a 6- × 40-mm angioplasty balloon (Admiral, Medtronic, Dublin, Ireland), and an 8- × 60-mm self-expandable stent was deployed across the lesion. Post-stenting dilation was conducted with an 8- × 40-mm angioplasty balloon (Figure 2).

**Figure 3:**
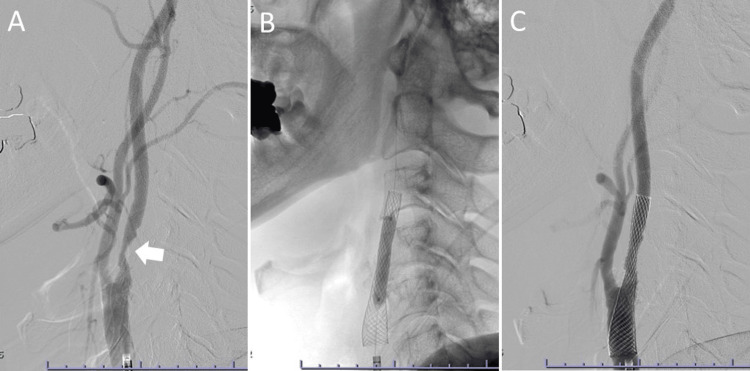
Right carotid artery stenting. (A) Stenosis in the right internal carotid artery is demonstrated (arrow). (B) Stent placement and postdilatation with a balloon. (C) The stenosis of the right internal carotid artery is shown to be improved

Subsequently, a 6-Fr guiding sheath (FUBUKI; Asahi Intecc Co., Ltd, Aichi, Japan) was advanced into the right common carotid artery via the right femoral artery. The stenosis was crossed and treated with a guidewire and a filter embolic protection device (FilterWire EZ; Boston Scientific, Natick, MA). Predilation was performed using a 2.5- × 40-mm angioplasty balloon (SHIDEN, Kaneka, Osaka, Japan) at 6 atmospheres (atm) for 30 seconds, and a 10- × 31-mm closed-cell stent (Carotid Wallstent, Boston Scientific) was deployed. Postdilation with a 4.5- × 30-mm angioplasty balloon (Sterling; Boston Scientific) at 6 atm for 10 seconds ensured adequate stent expansion (Figure 3). The contrast agent volume used was 28 mL. The patient was discharged home on the ninth day without complications.

**Figure 4:**
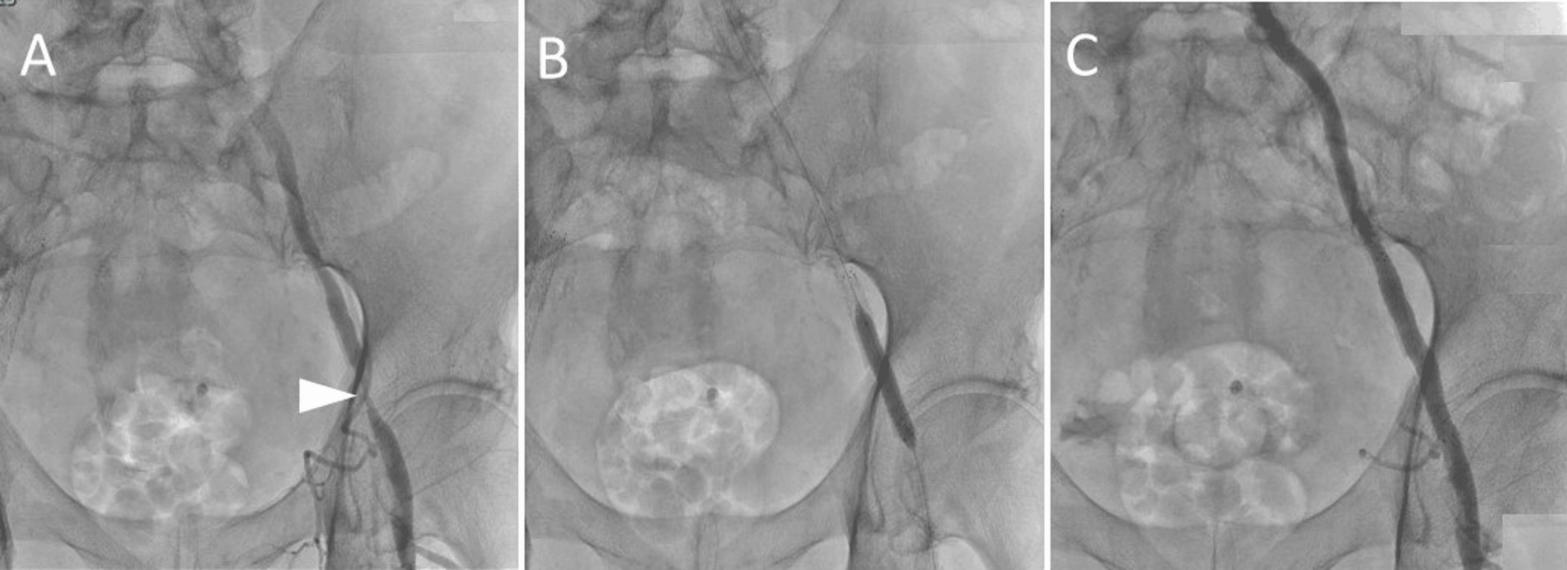
Left iliac artery percutaneous transluminal angioplasty with stenting. (A) Angiography shows stenosis in the left external iliac artery (arrowhead). (B) Stent placement and postdilatation with a balloon. (C) The stenosis of the left external iliac artery is shown to be improved

The second treatment was performed six months later. DSA via transfemoral access revealed 82% stenosis of the left external iliac artery. A 6.5-Fr short sheath was inserted into the right femoral artery, and a 150-cm guidewire was passed through the stenosis. Intravenous heparin was administered to achieve an activated coagulation time of approximately 300 seconds. Angioplasty of the lesion was performed using a 6- × 40-mm angioplasty balloon (Pacific, Medtronic), and an 8- × 60-mm self-expandable stent (EverFlex; EV3-Covidien, Plymouth, MN) was used across the lesion. Post-stenting dilation was performed using a 6- × 40-mm angioplasty balloon (Figure 4).

**Figure 5:**
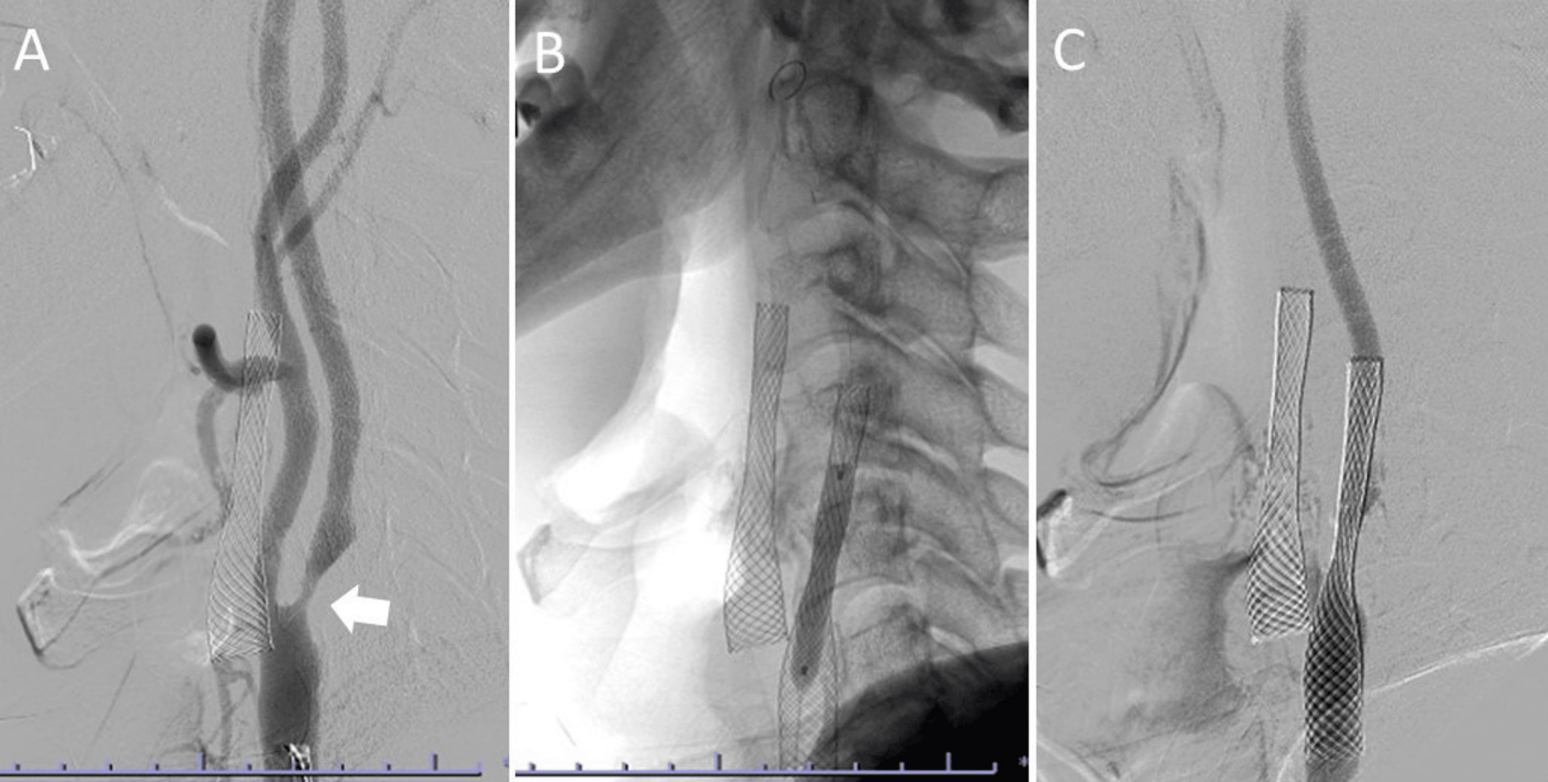
Left carotid artery stenting. (A) Stenosis in the left internal carotid artery is demonstrated (arrow). (B) Stent placement and postdilatation with a balloon are shown. (C) The stenosis of the left internal carotid artery is shown to be improved

Subsequently, a 6-Fr guiding sheath was advanced into the right common carotid artery via the right femoral artery. The stenosis was crossed and treated using a guidewire with a filter embolic protection device. Predilation was performed using a 2.5- × 40-mm angioplasty balloon (RapidCross, Medtronic) at 6 atm for 30 seconds, and a 10- × 31-mm closed-cell stent was used. Postdilation with a 4.5- × 30-mm angioplasty balloon at 6 atm for 10 seconds ensured adequate stent expansion (Figure 5).

The contrast agent volume used was 32 mL. The procedure proceeded uneventfully, and the patient was discharged on the ninth day with a Modified Rankin Scale score of 0. The ABPI improved from 0.50 to 0.74 on the right and from 0.41 to 0.59 on the left. The patient remained stable and asymptomatic at the six-month follow-up.

## Discussion

Simultaneous CAS and PTAS for the iliac artery effectively reduced the need for repetitive catheter punctures. The prevalence of carotid artery stenosis is high among patients with LEAD. Previous studies have shown that 14% and 19% of patients with LEAD have carotid artery stenosis of ≥70% and ≥50%, respectively [1,2]. The REduction of Atherothrombosis for Continued Health Registry reports that 25% of patients with LEAD have cerebrovascular disease [11]. The management of patients with both LEAD and carotid artery stenosis is unclear. Treatments should be prioritized based on the severity of each condition, with symptomatic diseases generally addressed initially.

In this case, the patient presented with both symptomatic LEAD and asymptomatic carotid artery stenosis. The risk of ipsilateral stroke or transient ischemic attack in patients with asymptomatic carotid artery stenosis is 0.5%-2% [12-14]. Treatment was considered necessary due to stenosis progression and high stroke risk. Additionally, we selected EVT for the iliac artery lesion because the patient had symptomatic LEAD and iliac artery lesion [15-17].

Our patient had an inaccessible carotid lesion via the transfemoral approach, requiring post-stenting antiplatelet therapy. This presented two options: performing carotid endarterectomy (CEA) followed by PTAS for the iliac artery or combining CAS with PTAS for the iliac artery. CAS was selected considering the patient’s history of coronary artery disease and the high risk associated with CEA.

Generally, CAS is performed via the transfemoral artery. However, limitations concerning puncture sites and treatment routes may arise in cases of systemic atherosclerotic disease. Although alternative puncture sites, such as the radial, brachial, and carotid arteries, have been reported [5-9], the transradial or transbrachial approach has been associated with higher technical failure rates compared with the transcarotid or transfemoral approaches [7]. Recently, transcarotid artery revascularization achieved favorable outcomes [6,8,9], but we were unfamiliar with this technique at the time. The advantages of our approach include the requirement for only one vascular puncture site and the feasibility of performing CAS via the femoral artery.

There are reports of simultaneous surgeries for multiple vascular lesions, such as carotid and coronary artery lesions. However, to the best of our knowledge, this is the first reported case of simultaneous CAS and PTAS for the iliac artery [18-20]. This case report presents a unique clinical scenario demonstrating the combined use of CAS and PTAS for the iliac artery as a viable and beneficial approach. The technique was feasible and advantageous for the surgeon, similar to the standard percutaneous transfemoral approach.

## Conclusions

This case report highlights the successful simultaneous execution of CAS and iliac artery PTAS in a patient with severe carotid and iliac artery stenosis. The advantages of our approach include requiring only one vascular puncture site and the feasibility of performing CAS via the femoral artery. This approach reduces the need for multiple interventions, minimizes procedural risks, and enhances patient recovery.
